# Randomized placebo-controlled trial on azithromycin to reduce the morbidity of bronchiolitis in Indigenous Australian infants: rationale and protocol

**DOI:** 10.1186/1745-6215-12-94

**Published:** 2011-04-14

**Authors:** Anne B Chang, Keith Grimwood, Andrew V White, Carolyn Maclennan, Theo P Sloots, Alan Sive, Gabrielle B McCallum, Ian M Mackay, Peter S Morris

**Affiliations:** 1 Child Health Division, Menzies School of Health Research, Charles Darwin University, Darwin, Northern Territory, Australia; 2 Queensland Children's Respiratory Centre, Royal Children's Hospital, Brisbane, Queensland, Australia; 3 Queensland Children's Medical Research Institute, The University of Queensland, Brisbane, Queensland, Australia; 4 Queensland Paediatric Infectious Diseases Laboratory, Royal Children's Hospital, Brisbane, Queensland, Australia; 5 Dept of Paediatrics, Townsville Hospital and School of Medicine, James Cook University, Townsville, Queensland, Australia; 6 Dept of Paediatrics, Royal Darwin Hospital, Darwin, Northern Territory, Australia

## Abstract

**Background:**

Acute lower respiratory infections are the commonest cause of morbidity and potentially preventable mortality in Indigenous infants. Infancy is also a critical time for post-natal lung growth and development. Severe or repeated lower airway injury in very young children likely increases the likelihood of chronic pulmonary disorders later in life. Globally, bronchiolitis is the most common form of acute lower respiratory infections during infancy. Compared with non-Indigenous Australian infants, Indigenous infants have greater bacterial density in their upper airways and more severe bronchiolitis episodes. Our study tests the hypothesis that the anti-microbial and anti-inflammatory properties of azithromycin, improve the clinical outcomes of Indigenous Australian infants hospitalised with bronchiolitis.

**Methods:**

We are conducting a dual centre, randomised, double-blind, placebo-controlled, parallel group trial in northern Australia. Indigenous infants (aged ≤ 24-months, expected number = 200) admitted to one of two regional hospitals (Darwin, Northern Territory and Townsville, Queensland) with a clinical diagnosis of bronchiolitis and fulfilling inclusion criteria are randomised (allocation concealed) to either azithromycin (30 mg/kg/dose) or placebo administered once weekly for three doses. Clinical data are recorded twice daily and nasopharyngeal swab are collected at enrolment and at the time of discharge from hospital. Primary outcomes are 'length of oxygen requirement' and 'duration of stay,' the latter based upon being judged as 'ready for respiratory discharge'. The main secondary outcome is readmission for a respiratory illness within 6-months of leaving hospital. Descriptive virological and bacteriological (including development of antibiotic resistance) data from nasopharyngeal samples will also be reported.

**Discussion:**

Two published studies, both involving different patient populations and settings, as well as different macrolide antibiotics and treatment duration, have produced conflicting results. Our randomised, placebo-controlled trial of azithromycin in Indigenous infants hospitalised with bronchiolitis is designed to determine whether it can reduce short-term (and potentially long-term) morbidity from respiratory illness in Australian Indigenous infants who are at high risk of developing chronic respiratory illness. If azithromycin is efficacious in reducing the morbidly of Indigenous infants hospitalised with bronchiolitis, the intervention would lead to improved short term (and possibly long term) health benefits.

**Trial registration:**

Australia and New Zealand Clinical Trials Register (ANZCTR): ACTRN12610000326099

## Background

Worldwide, bronchiolitis is the most common acute lower respiratory tract infection (ALRI) in infants[1-3]. In the Northern Territory (NT, Australia), ALRIs are the most frequent reason for hospitalisation of young children (aged <5-years). ALRIs are also the commonest cause of preventable deaths in Indigenous infants (5 times that of non-Indigenous)[4]. Of ALRIs, bronchiolitis (with or without pneumonia) is the most frequent reason for hospital admission in NT Indigenous infants aged under 12-months[5]. Despite this heavy burden of bronchiolitis in Indigenous infants, currently no prospective studies have been published. Our retrospective review of 101 infants hospitalised with bronchiolitis at the Royal Darwin Hospital (Darwin, Northern Territory) found that 33.7% of Indigenous infants were readmitted within six-months of discharge from hospital[6]. As most were retrieved from remote communities, the impact of the illness, its costs and social dislocation were likely to have been substantial.

Recurrent ALRIs are independently associated with the development of bronchiectasis[7] and reduced pulmonary function later in life[8]. Low birth weight and pre-existing small lungs are important determinants of future lung function, but there is increasing evidence that events in early life are at least equally important determinants of adult pulmonary dysfunction[8-10]. The first few years of life are the most critical period[11]. Thus events such as severe ALRIs during this critical period are likely to have long term effects.

Australia-wide, hospitalisation rates of respiratory disorders among Indigenous Australians are second only to those for renal dialysis[12]. Furthermore, we have previously documented that the severity of the hospitalised ALRI episode, as determined by oxygen requirement and duration of hospitalisation, was an independent risk factor for subsequent bronchiectasis[7]. In the Northern Territory (NT), bronchiectasis affects one in every 68 Indigenous children, far exceeding that of cystic fibrosis (CF) in non-Indigenous Australian children (1 in 2857)[13,14]. Thus, any intervention that reduces bronchiolitis severity and/or risk of readmission for respiratory illness in Indigenous infants may have both short term and potential long term benefits in our setting.

Bronchiolitis is characterised by extensive inflammation of the airways accompanied by increased mucous production and necrosis of airway epithelial cells. In paediatrics, bronchiolitis is a clinical diagnosis characterised by tachypnoea, wheeze and/or crepitations in infants following a preceding upper respiratory illness[1]. It is primarily caused by infection of the respiratory epithelial cells by a variety of viruses, especially respiratory syncytial virus (RSV). Other viruses (adenovirus, influenza, parainfluenza, human metapneumovirus, rhinovirus) are also implicated and increasingly new viruses are being detected in association with this illness[15]. In addition, *Mycoplasma pneumoniae *and Chlamydia species are recognised increasingly as important contributors to the development of chronic lung disease and altered lung maturation[16-19]. New treatable bacteria such as *Simkania negevensis *(a Chlamydia-like microbe) has been found in Canadian Inuit infants with bronchiolitis[20]. There are no published data on the nature or diversity of respiratory pathogens associated with bronchiolitis in Indigenous Australians infants.

Typically anti-microbials are not recommended in the routine management of bronchiolitis[1,21]. While there are two RCTs on macrolides for bronchiolitis, the single available RCT on a non-macrolide anti-microbial was a negative study[22]. However in our setting, there are several reasons why anti-microbials may reduce the morbidity of hospitalised Indigenous infants with bronchiolitis. Colonisation of nasopharynx with bacteria is a known risk factor for childhood pneumonia[23]. Indigenous infants not only have colonised nasopharynx very early in life (as early as aged 2-weeks), but the colonisation is also dense with common respiratory bacterial pathogens, notably *Streptococcus pneumoniae*, *Haemophilus infleunzae *and *Moraxella catarrhalis*[24]. Repeated micro-aspiration of nasopharyngeal secretions heavily laden with pathogenic bacteria during ALRI may overwhelm already compromised pulmonary defences, increasing the risk of a secondary pneumonia or other lower airway infection. Indeed, Indigenous infants are more likely to receive antibiotics for an episode of pneumonia diagnosed during an admission for bronchiolitis than non-Indigenous infants nursed in the same paediatric unit[6].

Macrolides are a class of antibiotics containing a macrocyclic lactone ring with excellent tissue penetration and antimicrobial activity against a broad range of gram positive and gram negative bacteria, including intracellular pathogens such as *Chlamydia*. Those with a 14- or 15-membered lactone ring also have several non-antimicrobial properties that have been studied extensively *in-vitro *and in experimental models and, to a lesser extent, in humans[25]. One of these effects is modulation of the immune response. The immune modulating properties of macrolides make them attractive candidates for treating inflammatory airways diseases. The two published[26,27] placebo-controlled RCTs on macrolides for RSV-bronchiolitis reported contradictory results. In a small Turkish study of 21 hospitalised infants with moderate to severe bronchiolitis, 3-weeks of daily clarithromycin significantly reduced severity of illness (oxygen use, hospital stay) and risk of hospital readmission for respiratory illness during the next 6 months[26]. However in another larger study involving 71 infants from the Netherlands, 3-days of azithromycin was not efficacious in infants hospitalised with bronchiolitis[27]. The difference in outcomes seen in the two trials may be related to the dose or length of treatment, chance, or heterogeneity of the study populations. Infants in Turkey are more likely to have concomitant bacterial infection compared to an affluent European group and moreover in Turkey, post-infectious childhood bronchiectasis remains an important health problem[28]. The populations also differed in age. The European study included infants up to 24-months of age, whereas the Turkish study involved only infants aged <7-months[26,27]. Clearly, a well designed RCT on the efficacy of macrolides to reduce the burden of bronchiolitis in a population at high risk of acute and chronic respiratory disease would be beneficial.

### Aims of the study

Our primary research question is: Amongst hospitalised Indigenous infants with bronchiolitis, does azithromycin (compared to placebo) improve clinical outcomes (length of stay in hospital and duration of oxygen supplementation)? Our primary hypothesis is that: The anti-microbial and anti-inflammatory properties of the macrolide, azithromycin, will improve the clinical outcomes of Indigenous infants hospitalised with bronchiolitis.

Our secondary aims are:

2. To determine the effect of azithromycin on readmissions into hospital within 6 months of treatment;

3. To assess the short-term impact of azithromycin on macrolide resistance patterns of respiratory bacterial pathogens in the nasopharynx; and

4. To describe the point prevalence and diversity of respiratory viruses, *Mycoplasma pneumoniae *and Chlamydia species using sensitive molecular diagnostic techniques.

## Methods

### Study design

**Figure-1:**
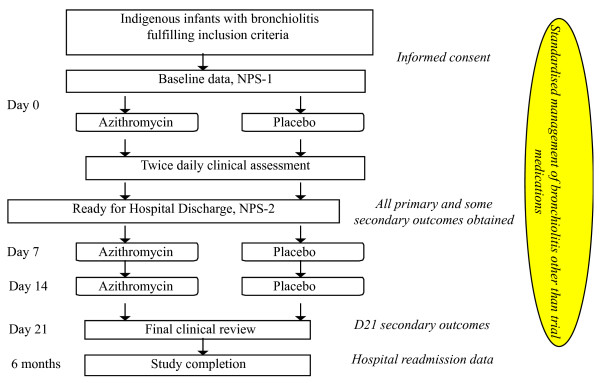
**Overall schematic study plan**.

We are conducting a parallel group, double-blind placebo RCT (with concealed allocation) to assess the impact of additional treatment with azithromycin in Indigenous infants admitted to two regional hospitals (Darwin, Northern Territory and Townsville, Queensland) with bronchiolitis. Our study plan is summarised in Figure 1.

### Eligibility

The inclusion criteria are:

1. Indigenous infants (aged ≤ 24-months) admitted to one of our hospitals (Darwin and Townsville) with a clinical diagnosis of bronchiolitis. In the absence of an international standardised diagnosis of bronchiolitis,[29,30] the accepted Australian clinical diagnosis is used (tachypnoea (respiratory rate ≥60/min in infants aged <2-months, ≥50/min if 2-12 months, and >40/min if 13-24 months), with wheeze or crackles);[31,32] and

2. Recruited and consented within 24-hours of presentation to the hospital for the illness.

Exclusion criteria: admission into intensive care, macrolide therapy contraindicated (e.g. liver dysfunction, hypersensitivity), presence of diarrhoea (stools of increased watery consistency and more than two stools above usual stooling frequency), received macrolides (in last 7-days), or clinical and radiological features consistent with a primary diagnosis of pneumonia,[33] at time of randomisation.

### Recruitment

At each site, the site-specific study nurse visits the wards twice daily to screen all newly admitted infants. A standardised collection form is used to collect clinical data (see below) and hospital outcomes associated with the bronchiolitis episode. All infants are managed according to a standardised protocol. This has been used at the Royal Darwin Hospital since 2008. The protocol outlines when supplementary oxygen is prescribed (Sp0_2_<94%) and reduced, and when nasogastric feeds or intravenous fluids are used. Enrolled infants may receive additional therapies (other than macrolides) at the discretion of the attending paediatrician.

### Intervention and follow up

If eligibility is fulfilled and after informed consent has been obtained, the infant is randomised to receive either a single, oral liquid dose of azithromycin syrup (30 mg per kg) or an equivalent volume of placebo. Medication is given within 24-hours of hospitalisation. Infants randomised to the intervention arm of the study will receive additional treatment doses of azithromycin syrup (30 mg per kg) on day-7 and day-14. Those randomised to the control arm receive an equivalent volume of the placebo syrup. The later doses will be either supervised by study nurses or administered by families with phone support on the day the medication is due. Final clinical follow up will occur in the local health clinic on day-21 (or closest available date from day 20 to 24).

### Randomisation, allocation and blinding

The randomisation sequence was computer generated and used permutated blocks (4 or 6 participants per block). The allocation sequence is concealed at all times throughout the study. The computer generation and allocation were performed by a statistician, external to the study team. Upon enrolment, an infant is assigned to the next number on the appropriate stratified list. Each unique number is assigned to one of the eight treatment alphabets (see below). Treatment groups are stratified by age (≤6 or >6 months), site (Darwin or Townsville) and requirement (yes or no) for oxygen at point of randomisation. The importance of excluding older children and stratifying at the 6 month age group is well described[30,34]. A placebo medication ensures that all children, carers, researchers, hospital staff, and clinic staff are blinded to treatment group until analyses of the data.

The placebo medication has been specifically manufactured by the Institute of Drug Technology (IDT) Australia Limited (Melbourne, Vic) which has a similar taste and colour to azithromycin. The azithromycin medications were repackaged by IDT. Thus both the azithromycin and placebo are in identical opaque bottles and sealed with an aluminium foil. For both, equal volumes of water are added using a syringe and needle by punching the seal.

Eight alphabets (N, O, P, Q, R, S, T, U) were used for the bottles of azithromycin and placebo medications (4 alphabets each). We used multiple alphabets rather than sequential numbers on the bottles to allow storage of extra bottles of trial medications in clinics for the day-7 and day-14 doses. This was necessary in the context of our study setting (children mainly from remote Indigenous communities), to enhance availability and administration of trial medications once the child has been discharged from the hospital.

### Data collection

All data are recorded on standardised forms. Demographic information (age, gender, region, birth details, smoke exposure, breast feeding, household size, etc) and medical history are obtained from the primary care-giver and the medical charts. The primary and secondary outcome measures (see below) are monitored twice daily until the hospital admission's end-point is reached (ready for respiratory discharge, defined as >16-hours without supplemental oxygen and infant is feeding well). Clinical assessment data include oxygen requirement and level, physiological measurements for clinical severity score (respiratory rate, accessory muscle use, degree of wheeze),[27] other physiological measurement (temperature, heart rate), requirement and duration of other therapies required (intravenous fluids, nutritional support, antibiotics), ear examination findings (signs of suppuration) and subsequent pneumonia (diagnosed by the independent treating 'blinded' paediatrician). In addition, the results of routine investigations (full blood count and chest radiographic findings) are recorded. On day-21, the presence of cough, wheeze and auscultatory abnormality on clinical review are documented. Adverse effects (vomiting, diarrhoea, rash) are also documented.

### Specimen collection

A single nasopharyngeal swab (NPS) specimen for respiratory virus and other potentially important respiratory pathogen (*M. pneumoniae, Chlamydia *spp) testing is collected from each subject at enrolment. In addition, NPS is repeated before hospital discharge for bacterial culture and antibiotic susceptibility testing, as per our laboratory research protocol (see below)[35,36].

### Laboratory methods

#### Bacteriology of NPS

Culturing, identifying and serotyping common respiratory bacteria from NPS is an established technique in our laboratory at Menzies in Darwin[35]. Swabs are stored in SMGGB at -80°C before being batch processed for typical respiratory bacterial pathogens, notably *H influenzae and *non-typeable *H influenzae*, *M. catarrhalis *and *S. pneumoniae*. Batches of swabs are thawed and 10 μL aliquots cultured overnight on selective media at 37°C in 5% CO_2_. Growth of *S. pneumoniae*, *H. influenzae *and *M. catarrhalis *is recorded and confirmed. Four isolates each of *S. pneumoniae *and *H. influenzae *and two isolates of *M. catarrhalis *per positive swab are tested for anti-microbial resistance and stored[35,37]. *S. pneumoniae *isolates are serotyped using the Quellung method (antisera from Statens Serum Institute, Denmark).

In addition to routine susceptibility testing using the calibrated dichotomous susceptibility (CDS) disc diffusion method, azithromycin minimum inhibitory concentration (MIC) will be determined using Etest (AB Biodisk, Sweden) if the azithromycin disc annulus is less than 6 mm. For *S. pneumoniae*, the penicillin MIC is determined for penicillin non-susceptible isolates (oxacillin and/or penicillin disc annulus <6 mm) and for *H. influenzae*, the ampicillin MIC is determined for isolates if the ampicillin disc annulus is less than 6 mm. Interpretive criteria (CSLI breakpoints) used for *S. pneumoniae *are penicillin non-susceptible MIC > 0.12 μg/mL, azithromycin resistant MIC ≥ 2 μg/mL, and for *H. influenzae*, ampicillin resistant MIC ≥ 4 μg/mL, azithromycin resistant MIC > 4 μg/mL. Beta-lactamase activity will be determined for *H. influenzae *and *M. catarrhalis *isolates.

#### Assessment for viruses and atypical bacteria

Our previous methods will be utilised[15,38,39]. NPS are frozen at -80°C. Upon thawing nucleic acids will be extracted from 0.2 ml of each NPS specimen using the High Pure Viral Nucleic Acid kit (Roche Diagnostics, Australia), according to the manufacturer's instructions. Mono-specific PCR and reverse transcriptase PCR (RT-PCR) method will be used to detect *Mycoplasma pneumoniae*, coronaviruses, bocavirus and human metapneumovirus (hMPV), whereas multiplex PCR and RT-PCR was used to detect adenovirus, parainfluenza (1, 2 3), influenza (A and B), and respiratory syncytial virus (RSV). All these methods have been previously validated in our viral laboratory at the Royal Children's Hospital, Brisbane.

### End point

Participation is complete when day-21 outcomes have been obtained. Other exit points are: intolerance to the trial medications requiring withdrawal from study (as deemed by the treating paediatrician who is not directly connected to the study team).

### Outcome measures

#### Primary outcomes

(i) Length of stay (LOS) for respiratory illness in hospital- defined as time from admission to time 'ready for discharge' for respiratory care. 'Ready for discharge' means normoxic (Sp0_2 _consistently >94% in air for >16-hrs) and feeding adequately; and (ii) Duration of supplemental oxygen required. 'Ready for discharge' for respiratory care differs from length of hospitalisation as discharge from hospital may be delayed because of social or transport factors especially in children from remote communities.

#### Secondary clinical outcomes

The major secondary outcome is readmission for respiratory illness (within 6-months of discharge from hospital). Minor outcomes during hospitalisation: clinical severity score,[27] additional use of antibiotics, and episodes of suppurative otitis media and development of pneumonia. The Day-21 outcomes are: presence of cough, wheeze, abnormal auscultatory chest signs and suppurative otitis media. We will also analyse all clinical outcomes in the following pre-determined sub-groups: (i) age ≤ 6-months; and (ii) presence of bacterial respiratory pathogens that are resistant to macrolide antibiotics.

#### Secondary laboratory outcomes

(i) identification of respiratory viruses and bacterial pathogens and (ii) antibiotic resistance to penicillin and macrolides.

### Sample size

We plan to enrol 200 Indigenous infants. In our retrospective study,[6] the mean LOS in Indigenous infants with bronchiolitis at RDH was 96 (SD 24) hours. The mean duration of supplemental oxygen requirement in Indigenous infants with bronchiolitis was 36 (SD 14) hours. For a mean difference of 24-hours in LOS between groups (power = 90%, α = 5%) the required sample size is 23 per group. The numbers to detect a 12-hour difference in supplemental oxygen requirement is 30 per group. These are large effect sizes but more conservative estimates than seen in the Turkish study[26]. In that study,[26] the difference between groups was 30-hrs for LOS and 31-hours for supplemental oxygen requirement. Assuming similar effects, a sample size of at least 100 in each sub-age group is also sufficient for an *a-priori *subgroup analysis based on age (power of 90% and 2-tailed α of 5%). If the effects are smaller, we will have an 80% power to detect a difference of 10-hours in LOS in all infants and an 80% power to detect a difference of 14-hours in LOS in the ≤6-months age group. We do not believe that smaller benefits than this would be sufficient to change clinical practice.

For the most important secondary outcome (readmission rate for a respiratory illness within 6-months of discharge), the power of our study to detect a reduction from 30% to 10% is 95% (5% significance). This is a large effect but consistent with the reduction described in the Turkish study (75% reduction)[26]. At 80% power we will be able to detect a reduction in readmission rates from 30% to 13%. For our other secondary outcomes, accurate sample size estimations are not possible given the lack of any relevant data.

### Statistical analysis and reporting

Data will be reported and presented in accordance with the updated CONSORT criteria[40]. Children will be analysed according to allocation status (regardless of subsequent management). An interim analysis is planned and the data safety and monitoring committee will determine if the study should be ceased should superiority of azithromycin be identified after 70% of sample size is achieved.

The primary outcomes (LOS and duration of supplemental oxygen requirement) will be compared between infants receiving placebo or azithromycin using unpaired Student's T-tests or Mann-Whitney tests (depending on normality of distribution). Although we expect randomisation to equally distribute potential confounding factors between each of the groups, we will examine the distribution of known confounders between groups (eg. birth weight, smoke exposure status in-utero and postnatal, breast feeding, etc). Should baseline data differ between groups, regression will be used to check that the primary outcomes are not affected by this chance finding. An *a-priori *sub-analysis will compare infants aged ≤ 6-months with those aged >6-months.

When examining the efficacy of azithromycin at reducing readmission rate for respiratory illness (Secondary Aim-2), the Odds Ratio (OR) between groups will be calculated. The OR will also be used to compare additional antibiotic use between groups. The number needed to treat (NNT) (for benefit), 95% CI will be described if any significant differences are found. If significant, NNT for harm will be calculated for adverse events. For Secondary Aim-3 (short-term impact of azithromycin on macrolide-resistance of pathogens in NPS cultures): the proportions of children with penicillin-non-susceptible *S. pneumoniae *and macrolide-resistant *H. influenzae *spp and *M. catarrhalis *before and after trial medications will be compared using ORs and 95% CI. Descriptive data will be utilised for Secondary Aim-4 (point prevalence of respiratory viruses and other respiratory pathogens).

### Ethical approval

The protocol has been granted full ethical approval from the respective Human Research Ethics Committees of all the participating institutions [Department of Health and Families (for Royal Darwin Hospital) and Menzies School of Health Research (Darwin), and the Townsville Hospital].

## Discussion

Acute lower respiratory tract infections are the commonest cause of hospitalisation and potentially preventable deaths in Indigenous infants[4]. Bronchiolitis in Indigenous infants is more severe than bronchiolitis in non-Indigenous infants[6]. There are higher readmission rates and an increased risk of ongoing respiratory morbidity (including chronic suppurative lung disease) in Indigenous infants[7,41]. This may be due to an increased likelihood of recurrent infections and virus-bacteria interactions from an early age (as early as aged 2-wks) along with heavy bacterial colonisation of the nasopharynx[36]. Despite bronchiolitis being the most common cause of ALRIs in infants resulting in hospitalisation, there are no published prospective studies of this illness in Australian Indigenous infants. Two small clinical trials have studied macrolides in bronchiolitis, but with contradictory results. Our population setting is more closely related to the Turkish study[26] where a beneficial effect for macrolides was shown. This is in contrast to the negative findings of the Dutch study[27] in an affluent urban setting.

Our proposed double-blind RCT will determine if azithromycin is an effective additional treatment in Indigenous infants hospitalised with bronchiolitis. It will also determine whether in the ensuing 6 months it will prevent hospital readmissions from respiratory illnesses, which potentially reduces the likelihood of chronic lung dysfunction[7] in this high-risk population. Azithromycin was chosen over other macrolides because of its prompt and potent anti-inflammatory effects as well as its 30-40 hours half-life in children, which permits once weekly dosing[42]. The possible anti-viral effects is also attractive[43]. An important safety component of the current study is to monitor for antibiotic resistance in potential respiratory bacterial pathogens colonising the nasopharynx.

For the first time in this population, we will determine the nature and diversity of respiratory viruses, *Mycoplasma, Chlamydia *and *Chlamydia*-like species in association with cases of bronchiolitis requiring hospital admission. The range of organisms includes newly discovered viruses,[44] and treatable bacteria that may contribute to chronic lung dysfunction. *M. pneumoniae *and *Chlamydia *species are increasingly recognised as important contributors to development of chronic lung disease and altered lung maturation[16-19]. Our study addresses a large clinical research gap for an important and common cause of hospitalisation in Indigenous infants. If the intervention is successful, it would lead to improved short term (and possibly long term) health benefits. Conclusive results would produce changes to evidence-based standard treatment guidelines in our region and those produced for similar populations nationally and internationally. Finally, the intervention would also offer the possibility of preventing (or reducing) the high rates of long-term respiratory dysfunction seen in Australian Indigenous children and adults.

### The rationale for our chosen outcome measures

Risk factors for bronchiectasis in Indigenous children include recurrent hospitalisation for ALRIs and severity of previous ARLIs (measured by duration of stay and requirement for oxygen supplementation during hospitalisation)[7]. In the context of the high burden of bronchiectasis in our setting, we considered that the most important outcomes are LOS, requirement for oxygen supplementation during hospitalisation and readmission within a 6-month period. Length of hospitalisation is a common outcome measure in studies on bronchiolitis. However in our setting, hospitalised children often come from remote comunities and may have multiple co-morbidities[33] that influences their discharge. Thus we used LOS defined in accordance with 'ready for respiratory discharge'.

### Limitations of our study

Our study only addresses infants hospitalised for bronchiolitis. The impact of variable presentation particularly that related to the potential influence of azithromycin's acute immune modulation effect is a limitation of our study. However our study design minimises the impact of variable presentation by: (a) standardising our inclusion criteria, (b) limiting enrolment to within 24 hours of hospitalisation; (c) adopting a strategy (double blind, placebo controlled, allocation concealed methodology) that would theoretically distribute any effect equally between groups. Additionally in the event that differences in baseline data between groups are found, we will use statistical methods (multivariate analysis) to adjust as required.

In summary, given the very high burden of bronchiolitis in Indigenous infants (the age when lung growth is most critical post-natally), and the association between ALRI and future lung dysfunction, our RCT on azithromycin in Indigenous Australian infants hospitalised with bronchiolitis has the potential to have both short term gains and a long-term benefit for reducing morbidity of respiratory illness.

## List of abbreviations

ALRI: Acute lower respiratory tract infection; LOS: Length of stay; MIC: Minimum inhibitory concentration; NPS: Naso-pharyngeal swab; RCT: Randomised controlled trial; RDH: Royal Darwin Hospital;

## Competing interests

The authors declare that they have no competing interests.

## Authors' contributions

AC conceived the study, and participated in its design and coordination and drafted the manuscript. PM, KG, TS, AW, AS, CM participated in its design, analysis plan and submission to the NHMRC. GM participated in initiating and running the project and IM in the viral analysis plan. All authors read and approved the final manuscript.
